# Elucidating the Role of circTIAM1 in Guangling Large-Tailed Sheep Adipocyte Proliferation and Differentiation via the miR-485-3p/PLCB1 Pathway

**DOI:** 10.3390/ijms25094588

**Published:** 2024-04-23

**Authors:** Yu Liang, Bishi Zhao, Yan Shen, Miao Peng, Liying Qiao, Jianhua Liu, Yangyang Pan, Kaijie Yang, Wenzhong Liu

**Affiliations:** College of Animal Science, Shanxi Agricultural University, Jinzhong 030801, China

**Keywords:** circTIAM1, miR-485-3p, PLCB1, adipocytes, proliferation, differentiation

## Abstract

Fat tissue—a vital energy storage organ—is intricately regulated by various factors, including circular RNA, which plays a significant role in modulating fat development and lipid metabolism. Therefore, this study aims to clarify the regulatory mechanism of sheep adipocyte proliferation and differentiation by investigating the involvement of circTIAM1, miR-485-3p, and its target gene PLCB1. Through previous sequencing data, circTIAM1 was identified in sheep adipocytes, with its circularization mechanism elucidated, confirming its cytoplasmic localization. Experimental evidence from RNase R treatment and transcription inhibitors highlighted that circTIAM1 is more stable than linear RNA. Additionally, circTIAM1 promoted sheep adipocyte proliferation and differentiation. Furthermore, bioinformatic analysis demonstrated a robust interaction between miR-485-3p and circTIAM1. Further experiments revealed that miR-485-3p inhibits fat cell proliferation and differentiation by inhibiting PLCB1, with circTIAM1 alleviating the inhibitory effect via competitive binding. In summary, our findings elucidate the mechanism through which circTIAM1 regulates Guangling Large-Tailed sheep adipocyte proliferation and differentiation via the miR-485-3p–PLCB1 pathway, offering a novel perspective for further exploring fat metabolism regulation.

## 1. Introduction

Sheep are economically important livestock that yield various valuable products, including meat, wool, leather, and milk. According to tail type, sheep can be divided into three types: fat-tailed sheep, fat-rumped sheep, and thin-tailed sheep. Fat-tailed sheep include long fat-tailed sheep and short fat-tailed sheep, mainly distributed in the northwest and northern regions of China. Due to human activities, the geographical distribution of sheep has changed [1]. The tail type differences resulting from this geographical distribution are mainly adaptations to the environment, a result of natural and artificial selection [2,3]. Sheep distributed in high-altitude areas have a higher proportion of energy metabolism-regulating genes [4], further demonstrating that sheep adjust their fat metabolism mechanisms to adapt to the environment. As the tail is the main site of fat storage in fat-tailed sheep, sheep with different tail types have become important animal models for studying fat deposition and energy storage [4]. For instance, in fat-tailed sheep, white adipose tissue is mainly found subcutaneously around the tail, with relatively low levels around the viscera and muscle tissues [4]. The fat tail serves as a significant energy reservoir, enabling these sheep to adapt to harsh environmental conditions such as droughts, extreme cold, and feed shortages [5]. The Guangling Large-Tailed sheep, as a type of fat-tailed sheep, has sparked the interest of researchers due to its significant adaptation to its environment. Despite being primarily bred for meat, the high tail-fat content, that can make up to 20% of the body weight, decreases the market value. This is attributed to consumer preference for leaner meat, thereby significantly affecting its economic value [6]. Therefore, studying the mechanism of fat deposition in sheep tails is important for improving production efficiency.

Fat deposition involves complex processes regulated by various transcriptional factors [7]. Circular RNAs (circRNAs) are noncoding RNAs characterized by a closed-loop structure, lacking 5′ caps and 3′ polyA tails. They exhibit structural conservation and stability, making them resistant to degradation [8]. CircRNAs are prevalent in eukaryotic organisms, primarily in the cytoplasm and extracellular vesicles, with a minority found in the nucleus [9]. Recent studies highlight their role in transcriptional regulation, alternative splicing, and chromatin looping [10,11,12]. Additionally, circRNAs function as molecular sponges, binding to miRNAs, influencing target gene expression [13]. For example, circSAMD4A serves as a sponge for miR-138-5p, regulating EZH2 expression and adipogenesis in individuals with obesity [14]. Similarly, CircBDP1 may regulate SIRT1 expression during bovine fat development by sequestering miR-204 and miR-181b [15]. Additionally, CircPAPPA2 acts as a competing endogenous RNA (ceRNA), sequestering miR-2366 to regulate GK expression, thereby inhibiting adipocyte differentiation [16]. Collectively, these findings suggest a pivotal role for circRNAs in sheep adipocyte development.

From prior sequencing data, circ-0000189 was identified within the sixth exon of the T-cell lymphoma invasion and metastasis 1 (TIAM1) gene, which was called circTIAM1. TIAM1 regulates the actin cytoskeleton and controls adipocyte formation [17]. Therefore, we hypothesized a significant role for circTIAM1 in adipocyte development. Additionally, we predicted a strong binding affinity between circTIAM1 and miR-485-3p. Studies show that miR-485-3p regulates TRIP6 expression, inhibiting cell proliferation [18]. Similarly, we predicted that phospholipase C beta 1 (PLCB1), a downstream target of miR-485-3p, regulates cyclin D3, CyclinE, and CyclinB1 levels. This promotes cell proliferation and 3T3L1 adipocyte differentiation [19]. With these findings, we propose circTIAM1 as a ceRNA in the miR-485-3p/PLCB1 pathway, regulating sheep adipocyte development.

Therefore, this study aims to elucidate the regulatory mechanism involving circTIAM1, miR-485-3p, and its target gene PLCB1 in Guangling Large-Tailed sheep adipocyte proliferation and differentiation. We specifically investigate how circTIAM1 upregulates PLCB1 by competitively binding miR-485-3p, thereby promoting sheep adipocyte proliferation and differentiation. The findings could offer important insights into the circRNA regulatory network in sheep fat deposition and provide valuable references for future investigations into the regulatory mechanisms underlying sheep fat metabolism.

## 2. Results

### 2.1. Characterization of circTIAM1 in Ovine Adipocytes

**Figure 1:**
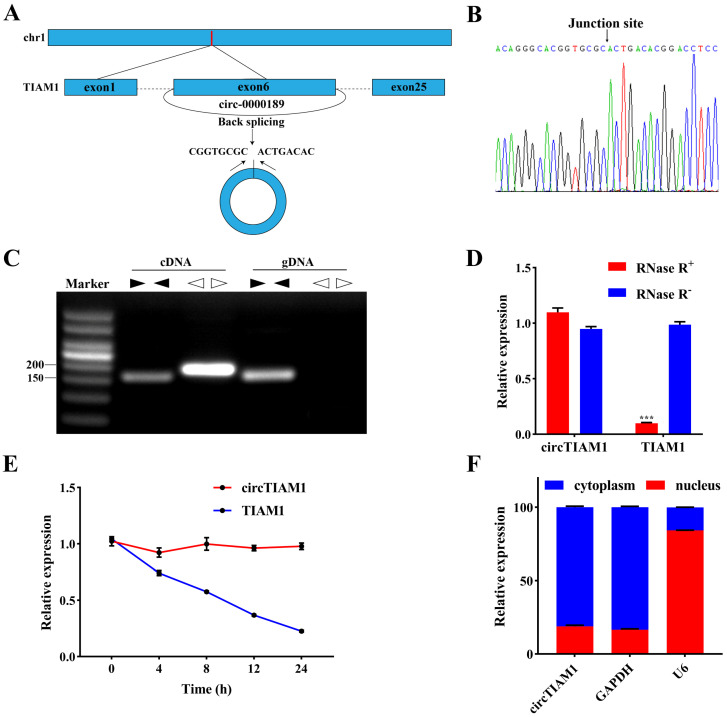
Characterization and identification of sheep circTIAM1. (**A**) CircTIAM1 formation via circular splicing of TIAM1. (**B**) Confirmation of circTIAM1 presence through Sanger sequencing. (**C**) PCR analysis using divergent and convergent primers in cDNA and gDNA. (**D**) CircTIAM1 and TIAM1 expression after RNase R treatment. (**E**) CircTIAM1 and TIAM1 expression level in sheep adipocytes following ActD treatment. (**F**) CircTIAM1 expression in nucleus and cytoplasm after nuclear-cytoplasmic fractionation. ***: *p* < 0.001.

Figure 1A illustrates the CircTIAM1 Circularization Scheme. Sanger sequencing (Figure 1B) confirmed the presence of circTIAM1 back-splicing products. Gel electrophoresis results demonstrated that divergent primers for circTIAM1 exclusively amplified circular products (168 bp) from cDNA, with no detection in genomic DNA (gDNA) (Figure 1C). However, convergent primers yielded amplification products (178 bp) for cDNA and gDNA (Figure 1C). Treatment of extracted RNA with RNase R showed no significant change in circTIAM1 expression (*p* > 0.05). However, TIAM1 expression significantly decreased (*p* < 0.05) (Figure 1D). Moreover, after treatment with actinomycin D (ActD-), a transcription inhibitor, circTIAM1, exhibited greater stability than TIAM1 (Figure 1E). Nuclear and cytoplasmic separation experiments revealed circTIAM1 localization in the cytoplasm (Figure 1F).

### 2.2. circTIAM1 Promotes Adipocyte Proliferation 

**Figure 2:**
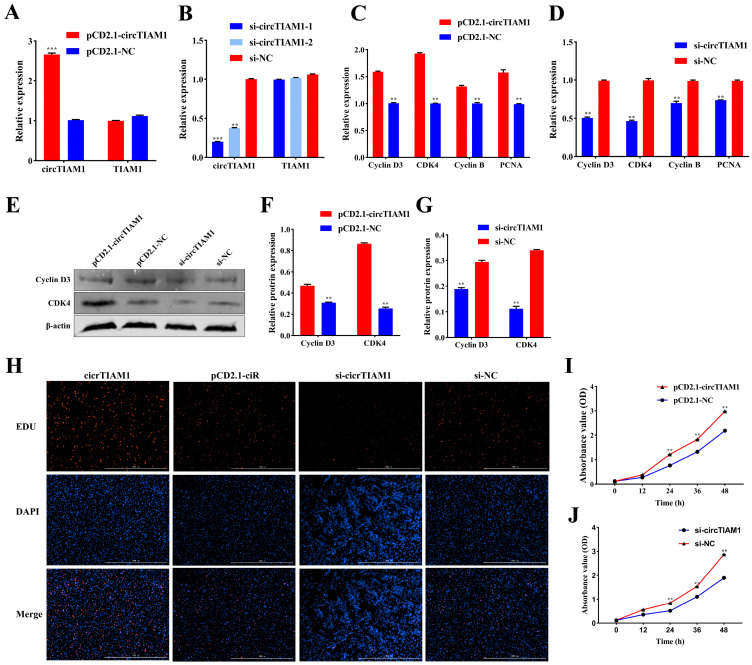
The effect of circTIAM1 on adipocyte proliferation. (**A**) circTIAM1 and TIAM1 expression after transfection of pCD2.1-circTIAM1 into adipocytes. (**B**) circTIAM1 and TIAM1 expression after transfection of si-circTIAM1 into adipocytes. (**C**) qRT-PCR analysis of cyclin D3, CDK4, cyclin B, and PCNA mRNA expression after pCD2.1-circTIAM1 transfection into adipocytes. (**D**) qRT-PCR analysis of cyclin D3, CDK4, cyclin B, and PCNA mRNA expression after si-circTIAM1 transfection into adipocytes. (**E**) Western blot analysis of cyclin D3 and CDK4 protein expression after circTIAM1 overexpression and interference. (**F**,**G**) Protein grayscale value analysis. (**H**) EdU analysis of adipocyte proliferation. (**I**,**J**) CCK-8 analysis of adipocyte proliferation. ** *p* < 0.01, *** *p* < 0.001.

To assess the effect of circTIAM1 on adipocyte proliferation, we transfected the circTIAM1 overexpression plasmid pCD2.1-circTIAM1 and interference plasmids si-circTIAM1-1 and si-circTIAM1-2 into sheep adipocytes. We observed a significant increase in circTIAM1 expression (*p* < 0.05) (Figure 2A). Si-circTIAM1-1 exhibited superior interference efficiency compared to si-circTIAM1-2 (*p* < 0.05) (Figure 2B). Therefore, it was selected for subsequent experiments. Additionally, qRT-PCR analysis revealed that pCD2.1-circTIAM1 upregulated the expression of Cyclin D3, cyclin-dependent kinase 4 (CDK4), Cyclin B, and proliferating cell nuclear antigen (PCNA) (*p* < 0.05) (Figure 2C). Conversely, si-circTIAM1 downregulated these expressions (*p* < 0.05) (Figure 2D). Furthermore, the protein expression levels of Cyclin D3 and CDK4 corresponded to the gene expression results (*p* < 0.05) (Figure 2E–G), suggesting circTIAM1 enhanced adipocyte proliferation. EdU staining revealed that circTIAM1 overexpression increased the production of new cells, promoting adipocyte proliferation (Figure 2H). Conversely, si-circTIAM1 exhibited contrasting outcomes (Figure 2H). Cell Counting Kit-8 (CCK-8) analysis revealed significantly higher absorbance values after 24 h of circTIAM1 overexpression than those in the control group (*p* < 0.05) (Figure 2I). This indicates enhanced cell activity by circTIAM1. However, interference with circTIAM1 led to opposing results (*p* < 0.05) (Figure 2J).

### 2.3. circTIAM1 Promotes Adipocytes Differentiation 

**Figure 3:**
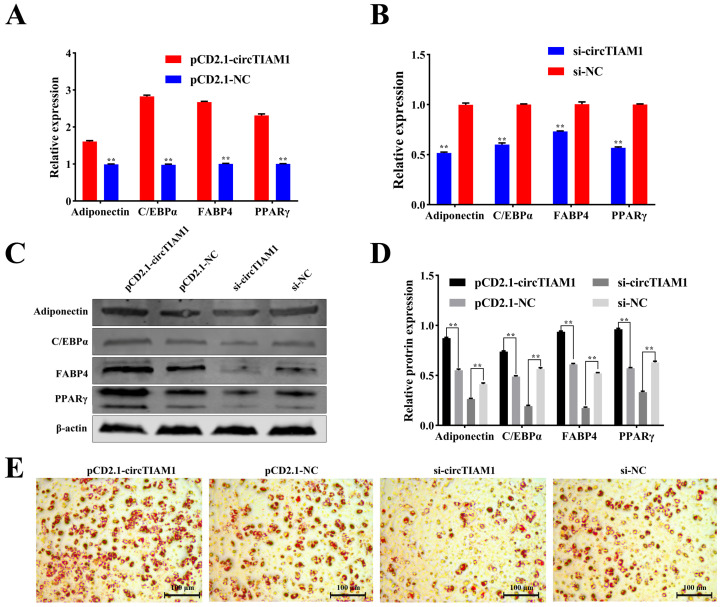
The effect of circTIAM1 on adipocyte differentiation. (**A**,**B**) qRT-PCR analysis of mRNA expression levels of Adiponectin, C/EBPα, FABP4, and PPARγ after pCD2.1-circTIAM1 and si-circTIAM1 transfection into adipocytes. (**C**,**D**) Protein expression levels of Adiponectin, C/EBPα, FABP4, and PPARγ in adipocytes analyzed via Western blot. (**E**) Oil Red O staining depicting circTIAM1-induced lipid droplet formation. ** *p* < 0.01.

To elucidate the effect of circTIAM1 on adipocyte differentiation, we transfected the circTIAM1 overexpression plasmid pCD2.1-circTIAM1 and interference plasmid si-circTIAM1-1 into sheep adipocytes. qRT-PCR analysis revealed that pCD2.1-circTIAM1 increased the expression of adipogenic marker genes Adiponectin, CCAAT/enhancer-binding protein α (C/EBPα), fatty acid binding protein 4 (FABP4), and peroxisome proliferator-activated receptor γ (PPARγ) (*p* < 0.05) (Figure 3A). However, si-circTIAM1 demonstrated opposite effects (*p* < 0.05) (Figure 3B). Furthermore, the protein expression levels of Adiponectin, C/EBPα, FABP4, and PPARγ were consistent with gene expression results (*p* < 0.05) (Figure 3C,D). Oil Red O staining of cells induced to differentiate for 8 days indicated increased lipid droplet formation with circTIAM1 overexpression and reduced lipid droplet formation upon circTIAM1 interference (Figure 3E). These findings suggest that circTIAM1 promotes adipocyte differentiation in sheep.

### 2.4. miR-485-3p Inhibits Adipocyte Proliferation 

**Figure 4:**
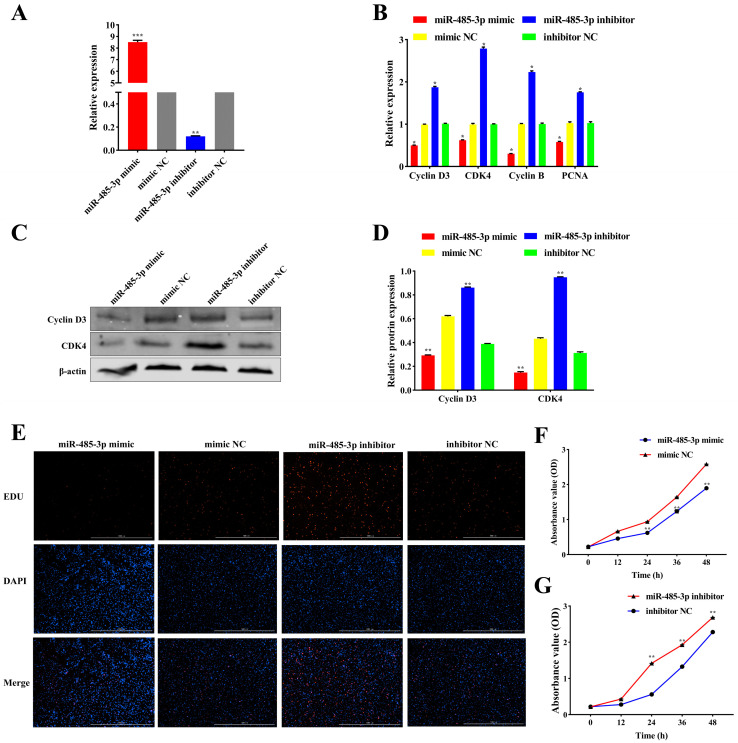
The effect of miR-485-3p on Adipocyte Proliferation. (**A**) miR-485-3p expression levels after transfection of miR-485-3p mimic and miR-485-3p inhibitor into adipocytes. (**B**) qRT-PCR analysis of Cyclin D3, CDK4, Cyclin B, and PCNA mRNA expression after overexpression and knockdown of miR-485-3p. (**C**,**D**) Western blot analysis of Cyclin D3 and CDK4 protein expression after miR-485-3p overexpression and knockdown. (**E**) EdU assay for adipocyte proliferation. (**F**,**G**) CCK-8 assay illustrating adipocyte proliferation. * *p* < 0.05, ** *p* < 0.01, *** *p* < 0.001.

To investigate the effect of miR-485-3p on adipocyte proliferation, we transfected the miR-485-3p mimic and miR-485-3p inhibitor into sheep adipocytes. The results demonstrated that the miR-485-3p mimic significantly upregulated the expression of miR-485-3p (*p* < 0.05) (Figure 4A). However, the miR-485-3p inhibitor significantly downregulated the expression of miR-485-3p (*p* < 0.05) (Figure 4A). Moreover, qRT-PCR analysis revealed that the miR-485-3p mimic suppressed the expression of Cyclin D3, CDK4, Cyclin B, and PCNA (*p* < 0.05) (Figure 4B). Conversely, the miR-485-3p inhibitor exhibited the opposite effect (*p* < 0.05) (Figure 4B). Furthermore, the protein expression levels of Cyclin D3 and CDK4 were consistent with the gene expression results (*p* < 0.05) (Figure 4C,D). These findings indicate that miR-485-3p suppresses adipocyte proliferation. EdU staining showed that the miR-485-3p mimic decreased newly generated cells, inhibiting adipocyte proliferation (Figure 4E). Conversely, transfection with a miR-485-3p inhibitor had the opposite effect (Figure 4E). CCK-8 results showed that after 24 h of miR-485-3p overexpression, absorbance values were significantly lower than those of the control group (*p* < 0.05) (Figure 4F), indicating inhibition of cell activity by miR-485-3p. Conversely, the knockdown of miR-485-3p resulted in opposite outcomes (*p* < 0.05) (Figure 4G).

### 2.5. miR-485-3p Inhibits Adipocyte Differentiation 

**Figure 5:**
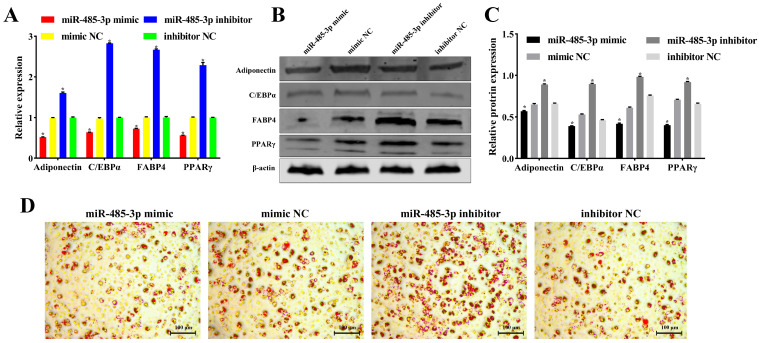
The effect of miR-485-3p on adipocyte differentiation. (**A**) qRT-PCR analysis of Adiponectin, C/EBPα, FABP4, and PPARγ mRNA expression levels after transfection of miR-485-3p mimic and miR-485-3p inhibitor into adipocytes. (**B**,**C**) Western blot analysis of Adiponectin, C/EBPα, FABP4, and PPARγ protein expression levels in adipocytes. (**D**) Oil Red O staining showing the inhibition of lipid droplet formation by miR-485-3p. * *p* < 0.05.

To examine the effect of miR-485-3p on adipocyte differentiation, we transfected the miR-485-3p mimic and miR-485-3p inhibitor into sheep adipocytes. qRT-PCR analysis revealed that the miR-485-3p mimic suppressed the expression of the adipogenic marker genes Adiponectin, C/EBPα, FABP4, and PPARγ (*p* < 0.05) (Figure 5A). However, the miR-485-3p inhibitor demonstrated opposing effects (*p* < 0.05) (Figure 5A). Moreover, protein expression levels of Adiponectin, C/EBPα, FABP4, and PPARγ aligned with gene expression results (*p* < 0.05) (Figure 5B,C). Oil Red O staining of cells induced for 8 days of differentiation revealed decreased lipid droplet formation with miR-485-3p overexpression. However, it increased lipid droplet formation upon miR-485-3p knockdown (Figure 5D). These findings suggest that miR-485-3p inhibits adipocyte differentiation in sheep.

### 2.6. PLCB1: A Target of miR-485-3p 

**Figure 6:**
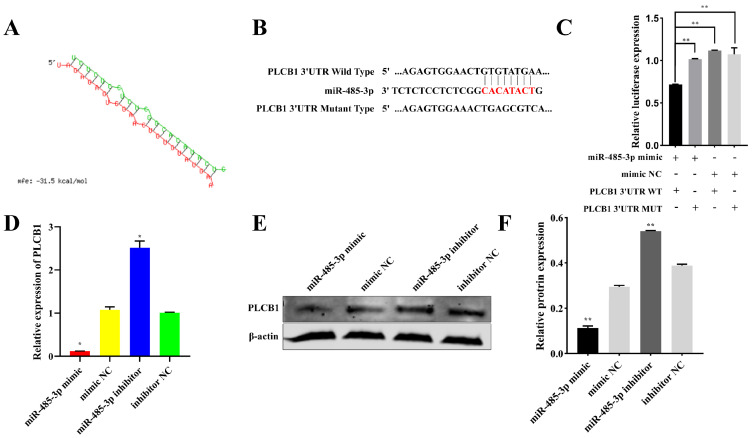
PLCB1; a target of miR-485-3p. (**A**) Predicted binding sites between miR-485-3p and PLCB1. (**B**) Binding and mutation sites of PLCB1 3′UTR with miR-485-3p. (**C**) Luciferase activity of wildtype and mutant PLCB1 3′UTR vectors cotransfected with miR-485-3p mimic or miR-485-3p NC into 293 T cells after 48 h. (**D**–**F**) Inhibition of PLCB1 expression by miR-485-3p at mRNA and protein levels in adipocytes. * *p* < 0.05, ** *p* < 0.01.

To elucidate the role of miR-485-3p in adipocyte proliferation and differentiation, we identified downstream regulatory genes. Using RNAhybrid, we predicted mRNA with miR-485-3p binding sites, discovering a binding site in the 3′UTR of PLCB1 (Figure 6A). Wild-type and mutant sequences containing the binding site were cloned into pmirGLO vectors to create PLCB1 3′UTR WT and PLCB1 3′UTR MUT vectors, respectively (Figure 6B). Co-transfection of these vectors with either the miR-485-3p mimic or miR-485-3p NC into 293T cells revealed that co-transfection of miR-485-3p mimic with PLCB1 3′UTR WT led to reduced luciferase activity than transfection with miR-485-3p NC (*p* < 0.05) (Figure 6C). However, co-transfection of PLCB1 3′UTR MUT with miR-485-3p mimic showed no significant effect on luciferase activity (*p* > 0.05) (Figure 6C), indicating a target relationship between miR-485-3p and PLCB1 3′UTR. Similarly, miR-485-3p suppressed PLCB1 expression at mRNA (*p* < 0.05) (Figure 6D) and protein levels (*p* < 0.05) (Figure 6E,F). These findings collectively indicate that PLCB1 is a target of miR-485-3p.

### 2.7. circTIAM1 Serves as a Molecular Sponge for miR-485-3p 

**Figure 7:**
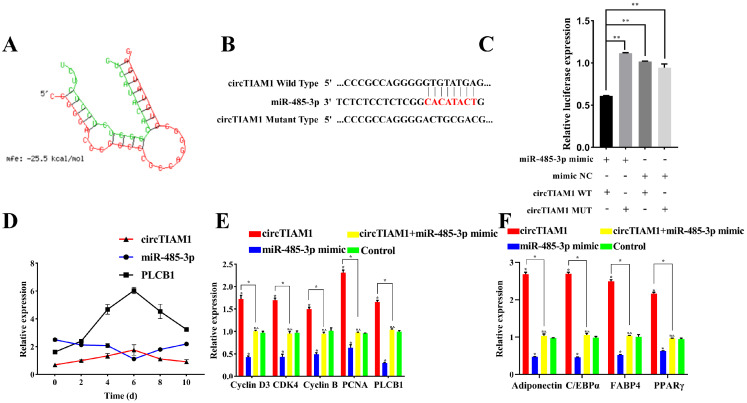
circTIAM1 as a ceRNA for miR-485-3p. (**A**) Predicted binding sites between circTIAM1 and miR-485-3p. (**B**) Binding and mutation sites of circTIAM1 with miR-485-3p. (**C**) Luciferase activity of wildtype and mutant circTIAM1 vectors cotransfected with miR-485-3p mimic or miR-485-3p NC into 293 T cells after 48 h. (**D**) circTIAM1, miR-485-3p, and PLCB1 expression patterns during adipocyte differentiation. (**E**) qRTPCR analysis of mRNA of cyclin D3, CDK4, cyclin B, and PCNA expression levels. (**F**) qRTPCR analysis of mRNA of Adiponectin, C/EBPα, FABP4, and PPARγ expression levels. * *p* < 0.05, ** *p* < 0.01.

Recent studies suggest that circRNAs can function as molecular sponges, competitively binding and participating in post-transcriptional gene regulation. Using RNAhybrid, we predicted the binding sites of circTIAM1 and miR-485-3p (Figure 7A). To confirm this hypothesis, we constructed wild-type (circTIAM1 WT) and mutant (circTIAM1 MUT) vectors containing the binding sites (Figure 7B). Co-transfection of these vectors with miR-485-3p mimic (or miR-485-3p NC) into 293T cells revealed that co-transfection of the miR-485-3p mimic with circTIAM1 WT significantly decreased luciferase activity compared with transfection with miR-485-3p NC (*p* < 0.05) (Figure 7C). Conversely, co-transfection of the circTIAM1 MUT with the miR-485-3p mimic demonstrated no effect on luciferase activity (*p* > 0.05) (Figure 7C). This suggests a target relationship between miR-485-3p and circTIAM1. Additionally, temporal expression analysis during sheep adipocyte differentiation over 10 days demonstrated that circTIAM1 and PLCB1 expression peaked on day 6 of adipocyte differentiation. However, miR-485-3p expression reached its lowest point (Figure 7D). These expression patterns suggest a negative correlation between circTIAM1 and miR-485-3p and between miR-485-3p and PLCB1. In summary, these findings suggest that circTIAM1 functions as a molecular sponge to bind miR-485-3p competitively.

We co-transfected the miR-485-3p mimic and pCD2.1-circTIAM1 into adipocytes to determine whether the regulatory effect of circTIAM1 on sheep adipocytes relies on miR-485-3p. Overexpression of miR-485-3p suppressed the expression of the proliferation marker genes Cyclin D3, CDK4, Cyclin B, and PCNA (Figure 7E, *p* < 0.05). However, co-transfection with circTIAM1 mitigated the inhibitory effect of miR-485-3p on adipocyte proliferation (Figure 7E, *p* < 0.05). Similarly, overexpression of miR-485-3p significantly decreased the expression of the differentiation marker genes Adiponectin, C/EBPα, FABP4, and PPARγ (Figure 7F, *p* < 0.05). This was reversed by co-transfection with circTIAM1 (Figure 7F, *p* < 0.05). Additionally, circTIAM1 treatment alleviated the effects of miR-485-3p on PLCB1 expression (Figure 7E, *p* < 0.05).

**Figure 8:**
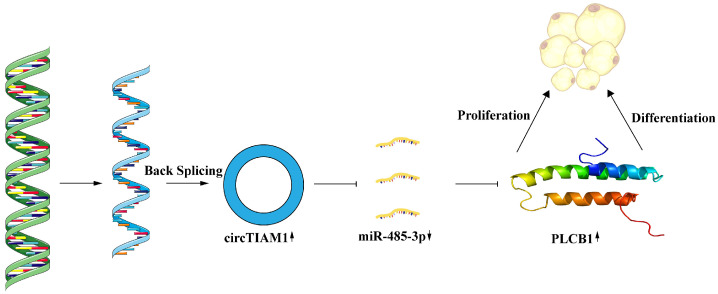
Mechanism of circTIAM1 regulation in Guangling Large-Tailed sheep adipocyte proliferation and differentiation through the miR-485-3p/PLCB1 Pathway.

These findings suggest that circTIAM1 enhances PLCB1 expression by competitively binding to miR-485-3p, consequently promoting Guangling Large-Tailed sheep adipocyte proliferation and differentiation (Figure 8).

## 3. Discussion

Fat tissue in animals functions as an energy storage site and plays a vital role in regulating metabolism [20], hormone secretion [21], immune function [22], and other essential physiological processes. Various non-coding RNAs (ncRNAs) regulate fat tissue formation in animals, with circRNA playing pivotal roles during adipogenesis [23,24,25]. Existing studies on circRNAs in sheep have predominantly centered on muscle development [26], hair follicle development [27], and reproductive performance [28]. Studies investigating the mechanisms through which circRNAs participate in adipocyte proliferation and differentiation remain limited. In this study, we discovered a circRNA uniquely expressed in sheep adipocytes, named circTIAM1, derived from the reverse splicing of the sixth exon of TIAM1. The identification and characterization findings revealed that the circularization mechanism of circTIAM1 predominantly occurs in the cytoplasm, aligning with its post-transcriptional regulatory role. ActD, as a transcription inhibitor, blocks new RNA synthesis, leading to the gradual degradation of already synthesized RNA. By collecting samples at different time points and measuring the levels of target RNA, decay curves for the target RNA can be established. Using these data, the half-life of the target RNA can be calculated, which is an important parameter for measuring RNA stability. Similar to other known circular RNAs [13], circTIAM1 exhibits higher stability compared to linear TIAM1 under RNase R and ActD treatments due to its closed-loop structure. This suggests its potential utility as a stable biomarker or therapeutic target for fat-related diseases [29]. Previous studies have highlighted the common correlation between the functions of circRNAs and their host genes [30]. TIAM1 regulates actin organization and fat cell formation [17]. Consequently, we hypothesized that circTIAM1 holds a considerable significance in adipocyte development. Our experimental results confirm that circTIAM1 stimulates the proliferation and differentiation of sheep adipocytes, aligning with previous findings. However, the specific mechanism underlying how circTIAM1 functions as a non-coding RNA remains unclear.

To explore the role of circTIAM1 as a non-coding RNA in regulating sheep adipocyte development, we conducted bioinformatics analysis and speculated on robust binding between miR-485-3p and circTIAM1. Further investigation into the role of miR-485-3p during adipocyte development demonstrated its inhibitory effects on the proliferation and differentiation of sheep adipocytes. Additionally, miR-485-3p inhibits cell proliferation by regulating TRIP6 expression, which is consistent with our findings [18]. The contrasting roles of miR-485-3p and circTIAM1 in sheep adipocytes indicate that circTIAM1 functions as a molecular sponge, modulating adipocyte development by sequestering miR-485-3p. However, the pathway through which miR-485-3p regulates adipocyte development remains elusive, highlighting the need for further research to elucidate these mechanisms.

Numerous studies have highlighted the role of miRNAs in gene expression level regulation through 3′UTR targeting of mRNA [31,32,33]. However, studies on their binding to the 5′UTR [34] and CDS sequences remain limited [35]. In this study, bioinformatics software (version 2.1.2) was utilized to predict a binding site between the seed sequence of miR-485-3p and the 3′UTR of PLCB1. Dual-luciferase reporter assays validated their target relationships, supported by several experiments, including qRT-PCR and Western blotting. These demonstrated a negative regulatory relationship between them. Additionally, PLCB1 has been shown to participate in the cell cycle progression by regulating the levels of various cell cycle proteins, thereby promoting cell proliferation. For example, PLCB1 can significantly increase the expression levels of Cyclin D3 and the percentage of S phase cells in the K562 human erythroleukemia cell line [36]. PLCB1 also modulates the expression of Cyclin E, which plays a critical role in the S phase of the cell cycle [37], and it can be involved in G2/M cell cycle progression [38]. During adipogenic differentiation, PLCB1 regulates the expression of cell cycle proteins cyclin D3 and cdk 4, which are essential for maintaining the differentiation state during both the early and late stages of mitosis [39]. Another study found that PLCB1 can enhance Cyclin D3 expression, activating PPARγ to promote adipogenesis [40]. This provides evidence elucidating how miR-485-3p inhibits the proliferation and differentiation of fat cells by suppressing PLCB1 expression.

CircRNAs commonly function as competitive endogenous RNAs, specifically binding to miRNAs and acting as molecular sponges to inhibit the effects of miRNAs on their target genes [41,42]. We constructed circTIAM1 WT and circTIAM1 MUT dual-luciferase reporter vectors to determine their target relationships. Co-transfection experiments involving circTIAM1 and miR-485-3p suggested that circTIAM1 mitigated the inhibitory effect of miR-485-3p on downstream PLCB1 through competitive binding, thereby regulating the proliferation and differentiation of sheep adipocytes. The expression patterns of circTIAM1, miR-485-3p, and PLCB1 during differentiation also partially demonstrated the role of circTIAM1 in regulating sheep adipocyte proliferation and differentiation via the miR-485-3p/PLCB1 pathway. However, miR-485-3p is not the sole target of circTIAM1. Moreover, it might regulate other target genes. This highlights the need for further exploration into the regulatory network of adipocyte development.

Advancements in the investigation of sheep adipocyte development regulatory mechanisms will enhance our understanding of circTIAM1, miR-485-3p, and PLCB1 regulatory networks. The integration of diverse experimental techniques and bioinformatics methods will facilitate the identification of more miR-485-3p target genes, potentially elucidating their functions and regulatory networks in adipocyte biology. Furthermore, our investigation into the role of PLCB1 in adipocyte proliferation and differentiation sheds light on its potential interactions with other signaling pathways. This offers a comprehensive understanding of its status and function in regulating fat cell metabolism. These studies contribute valuable insights into adipose tissue biology and disease development.

## 4. Materials and Methods

### 4.1. Ethical Approval Declarations and Sample Collection 

The methodologies employed in this study were conducted following the guidelines of the College of Animal Science and Veterinary Medicine at Shanxi Agricultural University (Jinzhong, China). All experimental Protocols were reviewed and approved by the institution (The Ethics Committee’s approval reference: SXAU-EAW-2022S. UV.010009).

This study employed Guangling Large-Tailed sheep as experimental animals, provided by Guangling County Sheep Farm in Datong, Shanxi. Three one-month-old Guangling Large-Tailed sheep were selected and euthanized by electric shock followed by exsanguination. Subsequently, tail fat cells were collected.

### 4.2. Cell Isolation and Culture 

Adipocytes were extracted from the tail fat tissues of 1-month-old lambs by mincing the tissues into small pieces with scissors. Type II collagenase (C8150, Solarbio, Beijing, China) was then employed to digest the fat tissue at 37 °C, 200 rpm for 30 min. The digestion process was halted by adding complete medium containing 10% fetal bovine serum (FBS; 04-001-1A, Biological Industries, Israel), 1% penicillin-streptomycin (10,000 U/mL of penicillin and 10,000 μg/mL streptomycin; 15140122; Gibco, Waltham, MA, USA), and Dulbecco’s Modified Eagle Medium (DMEM; C11995500BT, Gibco, Waltham, MA, USA). The digestion mixture underwent centrifugation at room temperature (1000 rpm for 10 min), and the resulting pellet was resuspended in a fresh complete medium. The suspension was then filtered through nylon meshes with apertures of 75 μm and 37.5 μm, respectively. The filtrate was cultured in a complete medium at 37 °C in a humidified atmosphere with 5% CO_2_. Medium changes occurred every 2 days. Once the precursor sheep adipocytes reached a fusion rate of 80–90%, the medium was replaced with a differentiation medium. This medium contained 89% DMEM, 1% penicillin-streptomycin, 250 μmol/L 3-isobutyl-1-methylxanthine (IBMX; I8450, Solarbio, Beijing, China), 500 μmol/L dexamethasone (ID0170, Solarbio, Beijing, China), and 8 μmol/L insulin (I8040, Solarbio, Beijing, China) for continued cultivation.

### 4.3. Vector Construction and Cell Transfection 

The complete circTIAM1 sequence was integrated into the pCD2.1-ciR vector (Geneseed, Guangzhou, China), while a vector lacking the circTIAM1 sequence served as a control. siRNAs targeting circTIAM1 splicing sites were synthesized by RiboBio (Guangzhou, China) (Table 1). Mimics and inhibitors of miR-485-3p were sourced from Sangon (Shanghai, China). The working concentration of mimics and inhibitors is 20 pmol/L. PLCB1 3′UTR WT, PLCB1 3′UTR MUT, circTIAM1 WT, and circTIAM1 MUT vectors were procured from Sangon (Table 2). When the density of sheep preadipocytes cultured in six-well plates reached 70%, cells were transfected using Lipofectamine 3000 (Invitrogen, Carlsbad, CA, USA) following the manufacturer’s instruction. After transfection for 6 h, the complete culture medium was replaced, and the cells continued to be cultured.

### 4.4. Cell Transfection 

Lipofectamine 3000 (Invitrogen) served as the transient transfection reagent.

### 4.5. RNase R Treatment 

The cells underwent treatment with 5 U/µg RNase R (R0301; Geneseed, Guangzhou, China), following the manufacturer’s instruction, and were then incubated at 37 °C for 15 min. Subsequently, the expression levels of circular and linear RNAs were assessed using qRT-PCR. 

### 4.6. Actinomycin D Assay 

Cells were exposed to 2 μg/mL ActD (MilliporeSigma, Burlington, MA, USA) and incubated for 4, 8, 12, and 24 h, respectively. The stability of the circular and linear RNAs was evaluated through qRT-PCR.

### 4.7. RNA and Genomic DNA Extraction and Quantitative Real-Time PCR

RNA was extracted from cells using RNAiso Plus (9108; Takara, Otsu, Japan) according to the instructions of the manufacturer. However, gDNA was extracted using DNAiso Reagent (9770; Takara, Otsu, Japan). Nuclear and cytoplasmic fractions were isolated using the PARIS kit (AM1921; Invitrogen, Thermo Fisher Scientific, Waltham, MA, USA). RNA quality was evaluated using the RNA 6000 Nano LabChip Kit and Bioanalyzer 2100 (Agilent Technologies, Beijing, China), while RNA integrity was confirmed through 1% agarose gel electrophoresis. Subsequently, RNA was reverse transcribed following the instructions of the manufacturer, using either the PrimeScript RT Reagent Kit with gDNA Eraser (Perfect Real Time; RR047A; Takara, Otsu, Japan) or miRcute Plus miRNA First-Strand cDNA Kit (KR211; Tiangen, Beijing, China). The qRT-PCR analysis was performed using TB Green Premix Ex Taq II Master Mix (RR820A; Takara, Otsu, Japan) or the miRcute Plus miRNA qPCR kit (FP411; Tiangen, Beijing, China) on a Bio-Rad CFX Connect Real-Time System (Bio-Rad, Hercules, CA, USA). Table 3 and Table 4 list the primer sequences employed in this study.

### 4.8. EdU and CCK-8 Analysis

The adipocytes were evenly seeded in 96-well plates. Cells in the logarithmic growth phase were assessed using the Cell-LightTM EdU DNA Cell Proliferation Kit (C10310-1; RiboBio, Guangzhou, China) in accordance with the instructions of the manufacturer. Furthermore, when cell confluency reached 60%, cells transfected at 0, 12, 24, 36, and 48 h received 10 μL of CCK-8 solution per well. Following a 2-h incubation, the absorbance at 450 nm was measured using a multifunctional microplate reader (Bio-Rad, Hercules, CA, USA) to generate cell proliferation curves.

### 4.9. Western Blotting 

Sheep adipocyte proteins were extracted using a protein lysis buffer with protease inhibitors (P6730; Solarbio, Beijing, China), phosphatase inhibitors (P1260; Solarbio, Beijing, China), and phenylmethylsulfonyl fluoride (PMSF; P8340; Solarbio, Beijing, China). These proteins were then separated via polyacrylamide gel electrophoresis and were transferred onto nitrocellulose filter membranes (HATF00010; Solarbio, Beijing, China). After washing with phosphate-buffered saline (PBS), membranes were blocked with 5% skim milk for 1 h. After removing the milk, they were incubated with primary antibodies overnight. Subsequently, the membranes were washed thrice with PBS-Tween (PBST) for 5 min each, followed by 2 h incubation with fluorescent secondary antibodies in the dark. After three 5-min washes with PBST, the membranes were imaged using the Odyssey Clx imaging system and quantified using ImageJ software version 1.8.0 (Bio-Rad, Hercules, CA, USA). Primary antibodies used for Western blotting were cyclin D3 (1:2000, 26755-1-AP, proteintech, Wuhan, China), CDK4 (1:2000, bs-0633R, Bioss, Beijing, China), PPARG (1:2000, 16643-1-AP, proteintech, Wuhan, China), C/EBPα (1:2000, 18311-1-AP, proteintech, Wuhan, China), FABP4 (1:1000, 12802-1-AP, proteintech, Wuhan, China), Adiponectin (1:1000, bs-0471R, Bioss, Beijing, China), PLCB1 (1:1000, 26551-1-AP, proteintech, Wuhan, China), and β-actin (1:1000, bs-0061R, Bioss, Beijing, China). The secondary antibody used was goat anti-rabbit IgG H&L/AP (1:10,000, bs-0295G-AP, LI-COR, Lincoln, NE, USA).

### 4.10. Oil Red Staining 

Differentiated adipocytes underwent three washes with pre-chilled PBS, and they were subsequently fixed with 4% paraformaldehyde at room temperature for 2 days. After fixation, the cells were stained with Oil Red O working solution for 30 min following the instructions of the manufacturer. Following destaining with 60% isopropanol for 60 s, the cells underwent three washes with PBS and were imaged under a microscope (Leica, Wetzlar, Germany).

### 4.11. Binding Relationship Prediction 

The binding sites between circTIAM1 and miR-485-3p, and between miR-485-3p and PLCB1, were predicted using RNAhybrid (https://bibiserv.cebitec.unibiele.org.de/rnahybrid), accessed on 1 December 2023.

### 4.12. Dual-Luciferase Reporter Assay 

The synthesized PLCB1 3′UTR WT, PLCB1 3′UTR MUT, circTIAM1 WT, and circTIAM1 MUT sequences were inserted into the double-luciferase reporter vector pmirGLO (Promega, Shanghai, China). The 293T cells were seeded in a 12-well culture plate. When the cells reached 70% confluence, they were transfected with Lipofectamine 3000. The test was divided into four groups with six repetitions per group. Co-transfected PLCB1 3′UTR WT and miR-485-3p mimic, PLCB1 3′UTR MUT and miR-485-3p mimic, PLCB1 3′UTR WT and miR-485-3p NC and PLCB1 3′UTR MUT and miR-485-3p NC. Co-transfected circTIAM1 WT and miR-485-3p mimic, circTIAM1 MUT and miR-485-3p mimic, circTIAM1 WT and miR-485-3p NC and circTIAM1 MUT and miR-485-3p NC. After co-transfecting these constructs with either miR-485-3p mimic or miR-485-3p NC into 293T cells, the relative expression ratio was determined. It compared firefly luciferase activity to the corresponding Renilla luciferase activity. This analysis was conducted 48 h post incubation according to the instructions of the manufacturer. 

### 4.13. Statistical Analysis 

Each experiment was performed with three samples, with each assessed thrice. Data analysis and visualization were conducted using GraphPad Prism software (version 8.0; GraphPad, San Diego, CA, USA). Student’s *t*-test was employed to assess the differences between the experimental and control groups, while a one-way analysis of variance was used for comparison between multiple groups. Data are presented as mean ± SEM, with a *p* < 0.05 considered statistically significant.

## 5. Conclusions

This study demonstrates that circTIAM1 promotes the proliferation and differentiation of Guangling Large-Tailed sheep adipocytes. Mechanistic studies have shown that circTIAM1 enhances PLCB1 expression by competitively binding to miR-485-3p, consequently promoting the proliferation and differentiation of Guangling Large-Tailed sheep adipocytes. The findings provide valuable insights into circRNA regulatory networks in sheep fat deposition, offering important references for future investigations into sheep fat metabolism regulation.

## Figures and Tables

**Table 1:** siRNA sequences targeting circTIAM1.

Name	Sequences (5′-3′)
si-circTIAM1-1	AGGGCACGGTGCGCACTGA
si-circTIAM1-2	TGCGCACTGACACGGACCT

**Table 2:** shRNA sequences targeting PLCB1 ^†^.

Names	Sequences (5′-3′)
sh-PLCB1 forward	GATCCGCACCCAAGGACCCTAAATTATTCAAGAGATAATTTAGGGTCCTTGGGTGCTTTTTTG
sh-PLCB1 reverse	AATTCAAAAAAGCACCCAAGGACCCTAAATTATCTCTTGAATAATTTAGGGTCCTTGGGTGCG
sh-PLCB1-2 forward	GATCCGCGTGGATTCATCTAACTATATTCAAGAGATATAGTTAGATGAATCCACGCTTTTTTG
sh-PLCB1-2 reverse	AATTCAAAAAAGCGTGGATTCATCTAACTATATCTCTTGAATATAGTTAGATGAATCCACGCG

^†^ Underscored sequences indicate enzyme sites.

**Table 3:** qPCR primers for mRNA application.

Name of Primers	GenBank Accession Number	Sequences (5′-3′)
circTIAM1 forward		GCCGTCAAGAACTTCCTGGTGC
circTIAM1 reverse		GACAGGAGGTCCGTGTCAGTGG
PPARγ forward	NM_001100921.1	ATCTTGACGGGAAAGACGAC
PPARγ reverse		AAACTGACACCCCTGGAAGAT
FABP4 forward	NM_001114667.1	AAACTGGGATGGGAAATCAACC
FABP4 reverse		TGCTCTCTCGTAAACTCTGGTAGC
Adiponectin forward	NM_001308565.1	ATCCCCGGGCTGTACTACTT
Adiponectin reverse		CTGGTCCACGTTCTGGTTCT
C/EBPα forward	NM_001308574.1	TCCGTGGACAAGAACAGCAA
C/EBPα reverse		TCATTGTCACTGGTCAGCTCC
β-Actin forward	NM_001009784.3	TGATGATATTGCTGCGCTCG
β-Actin reverse		GGGTCAGGATGCCTCTCTTG
miR-485-3p		GUCAUACACGGCUCUCCUCUCU
U6 forward	XR_003587591.1	CTCGCTTCGGCAGCACA
U6 reverse		AACGCTTCACGAATTTGCGT
PLCB1 forward	XM_042230393.1	TGGAGCTGGAGCAAGAATACC
PLCB1 reverse		AGAGCTGTGATTGCTGTCTTCA
Cyclin B forward	XM_060400336.1	CGTACTCCGTCTCCAGCC
Cyclin B reverse		AGCCAGTCAATCAGGATGGC
Cyclin D3 forward	XM_027958330.3	ACAGGCAGCATCGGAGCTGC
Cyclin D3 reverse		CTTTGGGCGCTGGGCTGGGA
PCNA forward	XM_004014340.5	ATCAGCTCAAGTGGCGTGAA
PCNA reverse		TGCCAAGGTGTCCGCATTAT
CDK4 forward	XM_012158548.4	CCAATGTTGTCCGGCTGATG
CDK4 reverse		CCTTGATCGTTTCGGCTGG

**Table 4:** Sequences of divergent and convergent primers for circTIAM1.

Name	Sequences (5′-3′)
circTIAM1 convergent	F: GGTAGCGAGTTTGCCGACAG
	R: AATTCTCATACACCCCCTGGC
circTIAM1 divergent	F: GCCGTCAAGAACTTCCTGGTGC
	R: GACAGGAGGTCCGTGTCAGTGG

## Data Availability

All data generated or analyzed during this study are included in this published article. The data that support the findings of this study are available from the corresponding author upon request.
